# Supplementary material for: Hepatic PEMT Expression Decreases with Increasing NAFLD Severity
Source: Int J Mol Sci. 2022 Aug 18;23(16):9296. doi: 10.3390/ijms23169296 (PMC9409182; doi:10.3390/ijms23169296)
Supplement: Supplementary file 1 [file ijms-23-09296-s001.zip › ijms-1860403-supplementary.pdf]

Table S1. Estimated posterior probability of lead SNP among significant *PEMT* variants

rsid	Variant Id	Position	Posterior_Prob
**rs4646385**	chr17_17545377_A_G_b38	17545377	0.287071
**rs12945144**	chr17_17530463_C_T_b38	17530463	0.0879812
**rs936108**	chr17_17536479_C_T_b38	17536479	0.0879215
**rs4646389**	chr17_17541035_C_A_b38	17541035	0.0856842
**rs4646387**	chr17_17541270_G_T_b38	17541270	0.0856842
**rs75459764**	chr17_17542223_T_C_b38	17542223	0.0847305
**rs77148834**	chr17_17542224_A_G_b38	17542224	0.0838336
**rs80097125**	chr17_17542232_C_T_b38	17542232	0.0847305
**rs12940673**	chr17_17542233_A_G_b38	17542233	0.0847305
**rs11348592**	chr17_17542237_TC_T_b38	17542237	0.0847305
**rs6502602**	chr17_17542245_A_T_b38	17542245	0.0847305
**rs6502603**	chr17_17542366_G_T_b38	17542366	0.0838336
**rs4646368**	chr17_17562144_G_A_b38	17562144	0.0793112
**rs4646356**	chr17_17567861_G_A_b38	17567861	0.0791296
**rs4646352**	chr17_17570539_C_T_b38	17570539	0.0793205
**rs76279970**	chr17_17542227_C_T_b38	17542227	0.0763488
**rs4646402**	chr17_17517873_G_C_b38	17517873	0.0963183
**rs4646388**	chr17_17541055_G_A_b38	17541055	0.0730155
**rs12941371**	chr17_17535004_G_A_b38	17535004	0.07126
**rs897450**	chr17_17572036_T_G_b38	17572036	0.0720769

Table S2. Allele frequency and test statistics of select *PEMT* SNPs in the bariatric surgery cohort

**Group**	**SNP**	**Position**	**A0**	**A1**	**A0 Freq (%)**		**Beta**	**se**	**P-value**
					**Fibrosis**	**Normal**			
**Total sample**	rs7946	17:17409560	T	C	83.3	69.1	0.881	0.508	0.083
	rs4646365	17:17467783	T	C	48.7	55.9	-0.176	0.394	0.656
	rs3760188	17:17487440	C	T	56.4	58.9	-0.237	0.411	0.563
**Postmenopausal women**	rs7946	17:17409560	T	C	85.7	75.0	0.350	1.411	0.804
	rs4646365	17:17467783	T	C	42.9	62.5	-1.512	1.153	0.188
	rs3760188	17:17487440	C	T	60.7	50.0	0.595	0.921	0.519

Table S3. Test statistics of *PEMT* variants with hepatic RNA expression in the bariatric surgery cohort							
**Group**	SNV	Position	A0	A1	beta	se	P-value
**Total Sample**	rs7946	17:17409560	T	C	0.045	0.151	0.766
	rs4646365	17:17467783	T	C	0.188	0.115	0.107
	rs3760188	17:17487440	C	T	-0.200	0.117	0.092
**Postmenopausal Women**	rs7946	17:17409560	T	C	0.071	0.258	0.787
	rs4646365	17:17467783	T	C	0.198	0.224	0.390
	rs3760188	17:17487440	C	T	-0.218	0.239	0.375

Table S4. *PEMT* allele frequencies in normal weight individuals from the UKB with NAFLD					
**SNP**	**A0**	**A1**	**A0 Freq (%)**		**P-value**
			**NAFLD**	**Normal Liver**	
**rs7946**	T	C	76.2	75.3	0.947
**rs1531100**	A	G	64.2	52.6	0.136
**rs4646365**	T	C	64.2	52.7	0.135
**rs12325817**	C	G	45.2	53.2	0.316
**rs3760188**	C	T	45.2	53.2	0.312
**rs4646343**	G	T	45.2	53.2	0.311
Frequency counts are from White British cohort (UKB) with BMI ≤25 kg/m², corresponding to 21 cases and 132,084 controls and adjusted for age, sex, diabetes status, and first ten PCs.					

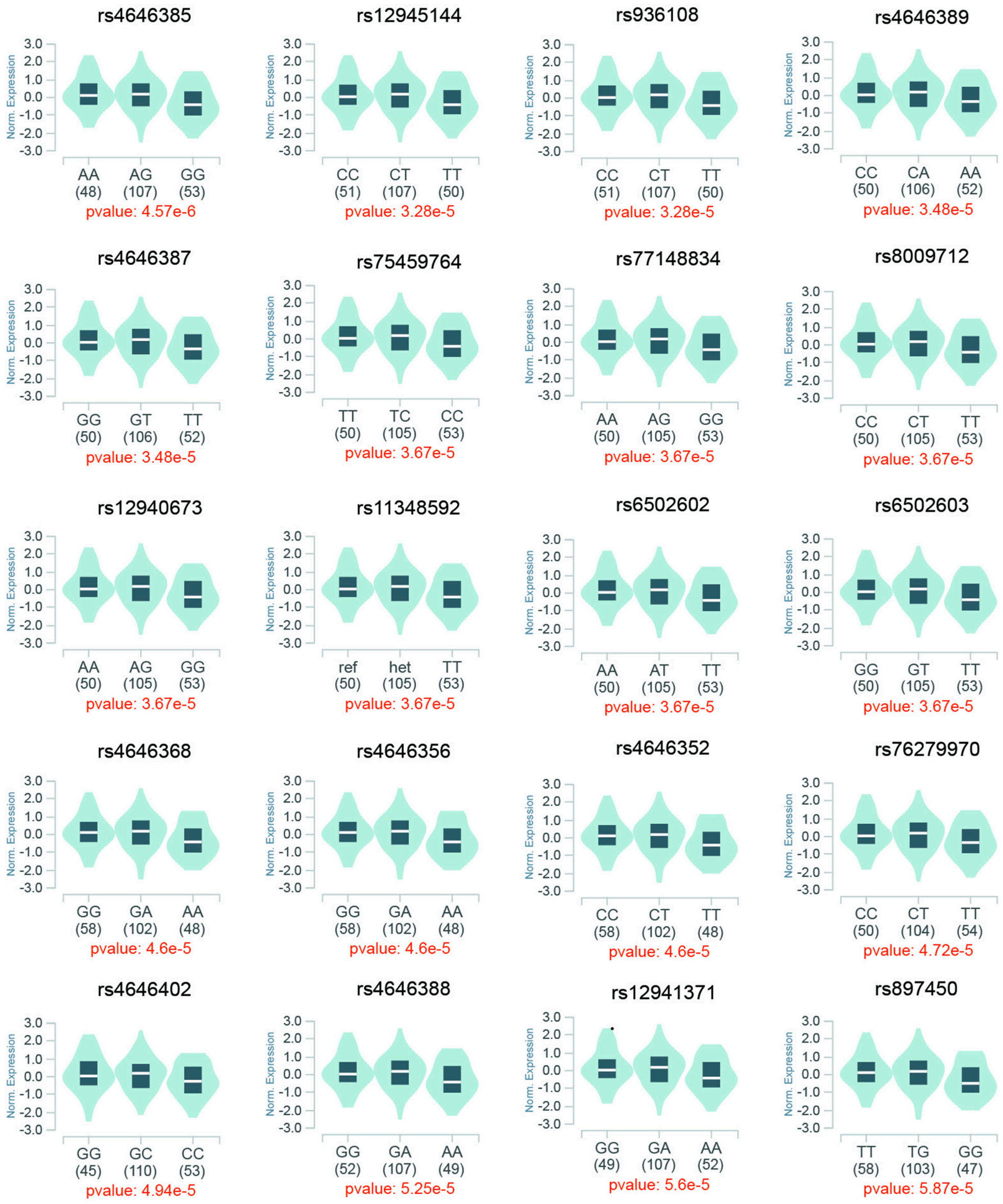

Figure S1. Allelic effects of SNPs significantly associated with hepatic PEMT expression. Violin plots and significance calculations were prepared from the GTEx database.

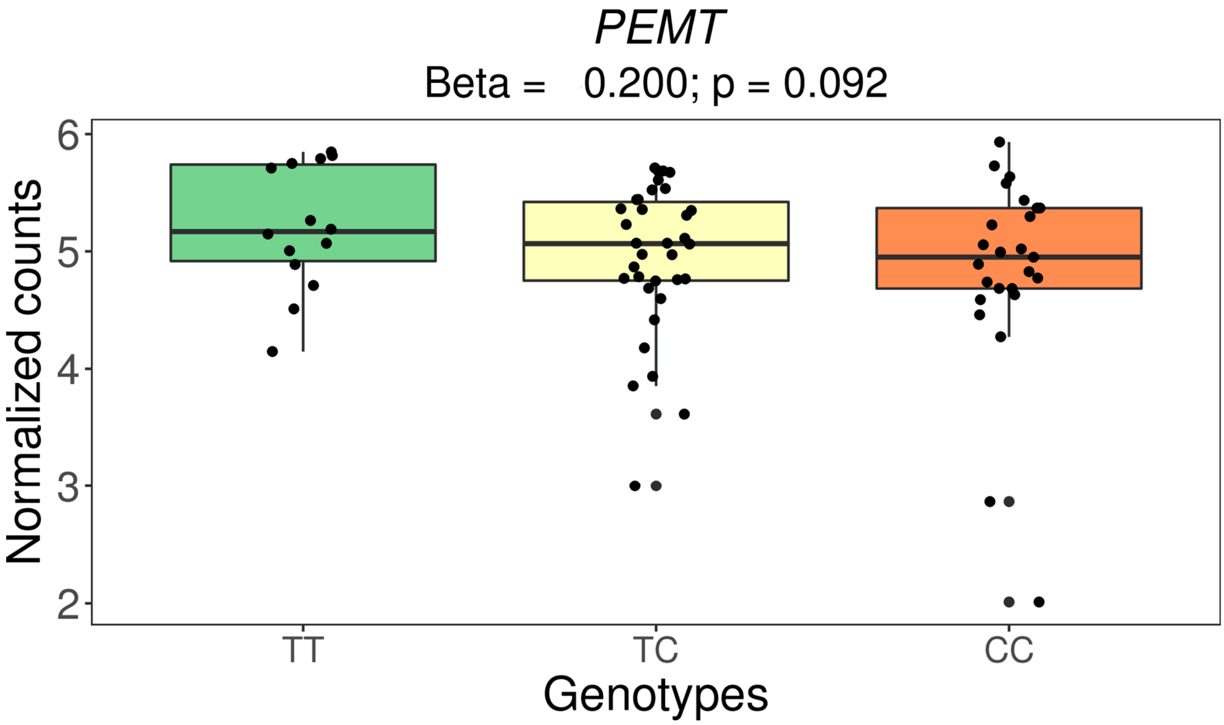

Figure S2. *PEMT* expression levels across rs3760188 genotypes.
